# Topogram and 3DCT geometry calibration for image-guided proton therapy with in-room CT-on-rails

**DOI:** 10.1016/j.phro.2025.100799

**Published:** 2025-06-24

**Authors:** Giovanni Fattori, Riccardo Via, Antony J. Lomax, Sairos Safai

**Affiliations:** a Center for Proton Therapy, Paul Scherrer Institut, Villigen, Switzerland; b Department of Physics, ETH Zürich, Switzerland

**Keywords:** CT-on-rails, In-room CT, Topogram imaging, Registration, Robotic patient positioning

## Abstract

In-room computer tomography (CT) on-rails scanners are available in proton therapy centers but often limited to control imaging, due to geometric distortion and lack of integration. We present a calibration method combining CT-on-rails with a robotic table to achieve sub-millimeter patient positioning accuracy. Geometric distortions in 3DCT and topogram images were corrected using affine models and validated with laser tracker data. Phantom experiments simulating clinical conditions showed errors below 0.15 mm for 3D/3D and 0.3–0.55 mm for 2D/3D registration, the latter using a novel algorithm for digitally reconstructed topograms. The workflow enables accurate positioning without isocenter verification, supporting high-precision radiotherapy.

## Highlights

• Corrected 3DCT and topogram distortions using affine transform models.
• Introduced fan-beam model for digitally reconstructed topogram generation.
• Achieved sub-millimeter accuracy in 3D/3D and 2D/3D patient positioning.
• Enabled high-precision treatments without verification imaging at the isocenter.

## 1 Introduction

In-room computer tomography (CT) scanners are available in several proton therapy centers for imaging patients in treatment position [1]. Their use is however often limited to control imaging, with treatment position verification at isocenter typically performed using on-board imagers. The main challenge with CT-based verification lies in calibrating the robotic table and CT coordinate systems to compute treatment positions from images acquired away from isocenter, while compensating for table sag.

Studies evaluating the performance of positioning in X-ray radiotherapy with CT-on-rails and pivoting table technology report sub-millimeter standard deviation errors in phantom measurements [2,3], but higher uncertainty in clinical settings [4,5], particularly along the scanner motion direction [6,7]. These errors often exceed the millimeter threshold and cannot be fully attributed to clinical variability alone, suggesting unresolved issues in imaging geometry calibration.

An early design for charged particle therapy used a vertical sliding CT to verify seated treatments at a fixed beam line [8], and was then followed by a gantry-based solution using a robotic table to reach a CT-on-rails installed at the side of the treatment room [9]. This configuration enabled clinical operation with positioning (random) errors below 2.5 mm [10] and supported adaptive workflows [11], though it relied on ad-hoc calibrations and procedures. Although similar room layouts have since been adopted by commercial vendors, in-room scanners have not been used for daily patient positioning due to the lack of full integration. This underutilization is largely attributable to methodological and quality assurance (QA) challenges, leading to inefficiencies and unnecessary imaging dose from repeated isocentric verification.

In this work, we present a methodology for calibrating in-room CT-on-rails for high-precision image guidance with robotic treatment tables. Our approach is compatible with current standards and treatment planning systems, modeling geometric distortions to enable accurate patient positioning. Additionally, we introduce a framework for fast computation of digitally reconstructed topograms. By addressing key integration challenges, this methodology provides a clinically deployable solution for IGRT using both 3DCT and 2D (topogram) imaging with an in-room CT-on-rails.

## 2 Materials and methods

### 2.1 Coordinate systems

Our room layout includes a proton gantry beamline [9], an in-room CT-on-rails (Sensation Open, Siemens Healthineers, Germany) and a robotic treatment table (Schär Engineering AG, Switzerland).

**Fig. 1:**
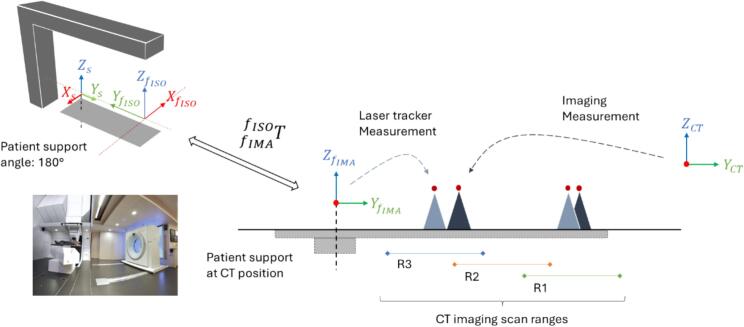
Coordinate systems (IEC-61217) for a Gantry beamline with remote imaging position and in-room CT-on-rails. The transform ${\;}_{f_{\mathrm{IMA}}}^{f_{\mathrm{ISO}}}T$ maps the fixed reference system at isocenter (f_ISO_) and its corresponding imaging position (f_IMA_). The treatment table is identified by the support frame of reference (s) and the CT scanner frame of reference (CT) is aligned to IEC standard. Two geometric objects are displayed as seen from laser tracker measurements relative to f_IMA_ (light blue) and imaging (dark blue), with increasing mismatch going from head (R1) to feet (R3) scan ranges. For illustrative purposes, the difference is exaggerated. (For interpretation of the references to colour in this figure legend, the reader is referred to the web version of this article.)

Fig. 1 shows the IEC-fixed (*f*) and patient support (*s*) coordinate systems (IEC-61217); a known transform ${\;}_{f_{\mathrm{IMA}}}^{f_{\mathrm{ISO}}}T$ maps the room frame of reference (FoR) at isocenter ($f_{\mathrm{ISO}}$) into its position at CT imaging ($f_{\mathrm{IMA}}$). The table bending correction enables alignment of any point in $f_{\mathrm{IMA}}$ to $f_{\mathrm{ISO}}$ with sub-millimeter accuracy (0.51 mm) using translational table compensation based on the payload and torque of the robot arms (Supplementary A, Fig. S4). Therefore, for the purpose of this study, it is relevant to map the in-room scanner FoR (CT) and $f_{\mathrm{IMA}}$, namely ${\;}_{\mathrm{CT}}^{f_{\mathrm{IMA}}}T$.

By fixing the table at a specific height and setting the CT FoR zero at the scanner’s parking position, the origin of the patient coordinates remains consistent across all images and has been conveniently oriented to align with IEC standard.

### 2.2 Modeling the 3DCT imaging coordinates distortion and frame of reference calibration

The transform ${\;}_{\mathrm{CT}}^{f_{\mathrm{IMA}}}T$ mapping the two reference systems, was defined as a six-degrees-of-freedom point-based registration. This was performed between image-derived coordinates from the CT scans of a geometric phantom (Supplementary B, Fig. S5), containing twelve 2 cm ceramic (Si_3_N_4_) spheres, and reference positions measured with a laser tracker (AT901-LR, Leica Camera AG, Germany) relative to $f_{\mathrm{IMA}}$. Images were acquired at three distances from the scanner FoR, defining regions R1-2-3 (Fig. 1), and each acquisition was repeated three times per region at 0.98 × 0.98 mm in-plane resolution and 1 mm slice thickness. Imaging coordinates were obtained via automatic segmentation [12] to evaluate reproducibility, while the average position in each region was used to optimize the transform.

Assuming linear longitudinal distortion, ${\;}_{\mathrm{CT}}^{f_{\mathrm{IMA}}}T$ was optimized by treating coordinate scaling as an independent free parameter for each of the three regions. Given a point ${\;}^{\mathrm{CT}}P$ in the CT coordinate system, the distortion compensated position ${\;}^{\mathrm{CT}}P^{\prime }$ is computed by applying an affine transform (*K*) scaling the IEC-Y-coordinate:

(1) $$\begin{array}{c} {\;}^{\mathrm{CT}}P_{Y}^{\prime }=K{*}^{\mathrm{CT}}P_{Y}=\left[\begin{array}{cccc} 1 & 0 & 0 & 0\\ 0 & \left(1+m\right) & 0 & c\\ 0 & 0 & 1 & 0\\ 0 & 0 & 0 & 1 \end{array}\right]{*}^{\mathrm{CT}}P_{Y} \end{array}$$

where m and c are the slope and intercept of the linear correction.

CT images of a simpler phantom with two spheres (Supplementary B, Fig. S5) were acquired for validation, comparing laser measurements with their distortion-compensated image coordinates.

### 2.3 Modeling the CT topogram images coordinates distortion

Topograms are radiographic images at the scanner center of rotation, generated by a fan-beam projection from a source moving along the longitudinal axis. Geometric distortion was calibrated by comparing the $Y_{\mathrm{CT}}$ of fiducials in topograms with their corresponding positions in 3DCT images, which had been corrected for longitudinal distortion (Eq. (1), and served as the reference. Thirteen 2 mm CT-dots (Beekley 119) were placed on foam blocks to pseudo-randomly sample the imaging field-of-view (FoV). Topogram images were acquired for lateral (LAT) and posterior-anterior (PA) projections at source angle (α) 90° and 180° around the $Y_{\mathrm{CT}}$ axis (Fig. 1), using both small and large FoV with in-plane resolutions of 1 mm and 2 mm. Two datasets (D1 and D2) including 3DCT, were collected approximately one year apart, with three repetitions per set.

Fiducials were manually localized in all 2D and 3D datasets to assess reproducibility, model distortion, and calibrate a projection method based on Siddon-Jacobs algorithm [13] for generating digitally reconstructed topograms (Supplementary C, Fig. S6) [14].

### 2.4 Calculation of treatment table position from in-room CT-on-rails imaging

The treatment table position is determined by registering the patient’s anatomy from planning images (pCT) to the in-room CT images (${\;}_{\mathrm{pCT}}^{\mathrm{CT}}T_{\mathrm{Patient}}$). This allows mapping the plan isocenter (${\;}^{\mathrm{pCT}}ISO$) into the in-room scanner FoR as follows:

(2) $${\;}^{\mathrm{CT}}ISO{=}_{\mathrm{pCT}}^{\mathrm{CT}}T_{\mathrm{Patient}}{*}^{\mathrm{pCT}}ISO$$

Using the scanner calibration transform ${\;}_{\mathrm{CT}}^{f_{\mathrm{IMA}}}T$ (Section 2.2), the isocenter position in CT coordinates is then mapped to the fixed reference at imaging position ($f_{\mathrm{IMA}}$). Due to geometric distortion, the CT coordinates must first be corrected using modality-specific models:

(3) $${\;}^{f_{\mathrm{IMA}}}ISO{=}_{\mathrm{CT}}^{f_{\mathrm{IMA}}}T{*}^{\mathrm{CT}}ISO\prime$$

Clinical conditions were simulated using a head phantom with a cranial fiducial sphere representing the isocenter (Supplementary B, Fig. S5). A pCT was acquired at 0.98 mm in-plane resolution and 1 mm slice thickness, followed by a displacement of approximately 1 cm laterally/vertically and 2 cm longitudinally. Volumetric 3DCT and topogram image pairs were acquired to assess the registration accuracy against laser tracker measurements.

Topogram-based 2D/3D registration was performed using Mattes mutual information (50 bins) with a Nelder-Mead optimizer (simplex delta = 2, tolerance = 0.1) [14]. Rigid 3D/3D registration was executed in Velocity (Varian Medical Systems, Palo Alto, CA, v.4.1). In both, only translational offsets were computed, consistent with the facility’s table control capabilities.

## 3 Results

### 3.1 3DCT imaging coordinates distortion and calibration

**Fig. 2:**
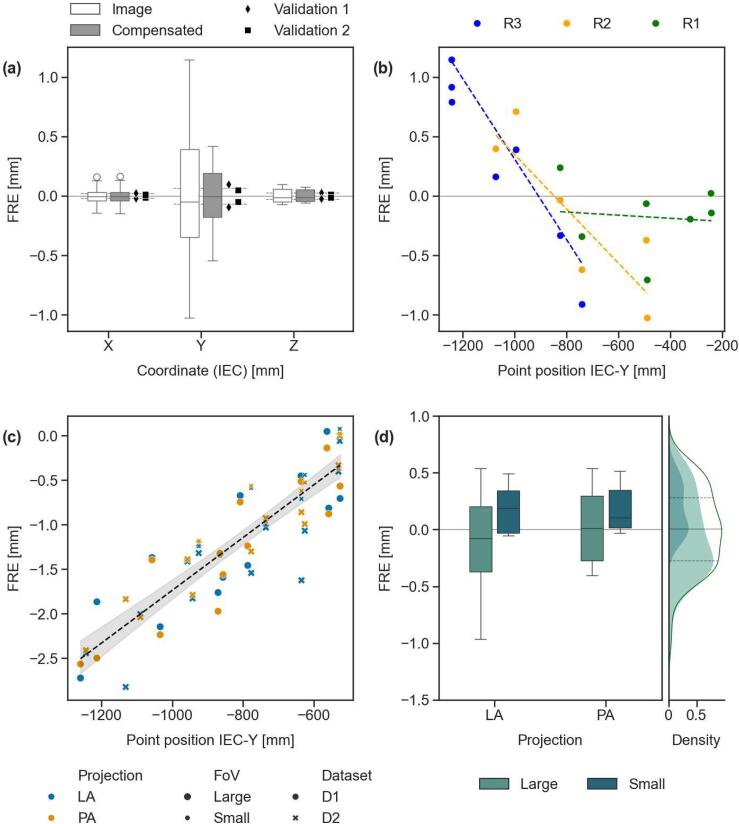
(a) Fiducial registration error (FRE) distributions for raw (image), distortion-compensated, and validation 3DCT datasets. The 95 % confidence interval of imaging point precision (deviation from mean position across repeated scans) is shown as reference. (b) FRE between reference and imaging positions in 3DCT for scan ranges R1–R3 due to image distortion. (c) Distance of topogram image points from reference as a function of their IEC-Y position, across datasets I and II, projection angles (LAT and PA), and FoVs (Large and Small). Linear fit shown with dashed line and 95 % confidence interval. (d) Topogram point error distributions grouped by FoV, with cumulative density for the full dataset. Mean and interquartile range (25^th^–75^th^ percentiles) indicated by dotted lines.

CT imaging reproducibility was high, with 95 % quartile range of the distance from the mean below 0.2 mm (Supplementary D). The use of calibrated distortion correction improves the ${\;}_{\mathrm{CT}}^{f_{\mathrm{IMA}}}T$ fiducial registration error (FRE) RMSE, from 0.59 mm (Fig. 2a, ‘Image’ data) to 0.27 mm after correction (Fig. 2a, ‘Compensated’ data and Supplementary E, Fig. S8). Distortion increased as the imaging range moved farther from parking position (Fig. 2b).

The correction parameters for the three regions, associated to their respective scan range center, were fit with a second-order polynomial curve (RMSE ≪ 1e-5) to interpolate between the calibrated points. The FRE for two validation scans centered at 984 mm and 1015.5 mm from the CT-FoR remained below 0.2 mm, a value consistent with image reproducibility (Supplementary D).

### 3.2 CT topogram images coordinates distortion

CT-dots reproducibility, measured as the deviation from their mean position in repeated scans, was as low as 0.5 mm (95 % interval), with comparable results between 3DCT and topograms (Supplementary D). Slight improvements were observed with smaller FoVs, and higher in-plane resolution.

Topogram distortion was modeled using Huber L2-regularized linear regression (RMSE: 0.33 mm) using data collected under different projections, FoV, and acquisition dates (Fig. 2c). Residuals from the fit were within ± 0.25 mm of the corresponding 3DCT points for 50 % of the data, and all within ± 1 mm. Overall, errors had a median of 0.01 mm and an interquartile range of 0.56 mm (Fig. 2d).

### 3.3 Calibration of topogram imaging projections

LAT and PA projections, each with small and large FoV, were calibrated individually. The RMSE of calibration points was below 0.1 and 0.3 mm for topograms acquired with small or large FoVs, respectively. Generally, the source angle deviated by less than 1° from the nominal value, and all others distances were within 1 cm of the design specifications (Supplementary F).

### 3.4 Clinical positioning simulation

An initial automatic 6-DoF registration on the phantom head region of interest was followed by a 3-DoF manual adjustment, yielding errors below 0.2 mm along individual directions using 3DCT positioning images. With topograms, an initial manual registration of the isocenter sphere was followed by automatic registrations focusing on the phantom head, excluding the isocenter sphere. Discrepancies were below 0.3 mm for higher-resolution images but reached up to 0.55 mm at larger FoVs (Supplementary G).

## 4 Discussion

This study addresses the harmonization of imaging coordinates between an in-room CT-on-rails and a robotic treatment table for image guidance in proton therapy, incorporating image distortion corrections to compute treatment table positions from daily scans. We demonstrated that, with proper calibration, CT-on-rails can meet sub-millimeter accuracy requirements for positioning imagers and be used without additional isocentric verification.

Unlike on-board imagers or C-arm-based CBCT [15], on-rails CT imaging requires the table to be in a fixed position, making it necessary to map CT coordinates to the room system. Robotic tables add complexity, as unlike simpler pivoting [16] or pantographic solutions [17], their bending differs between imaging and treatment positions. As a result, system calibration must account for both geometric distortions in imaging and table sag. The proposed method is broadly applicable and demonstrates theoretical accuracy below 0.3 mm for common topogram settings, and lower with 3DCT; a value that adds on robot mechanical precision, which is specific to the manipulator design (Supplementary A, Fig. S4).

We also explored topogram imaging, particularly suitable for sites near bony structures, as well as for verifying extremities and the cranio-spinal axis. Imaging the entire patient in a single scan eliminates the need for multiple adjacent verifications, a common limitation of gantry-mounted X-ray systems due to their restricted field of view [18,19]. It also reduces the daily 3DCT usage which is especially important for pediatric patients to minimize radiation exposure. Unlike physics-based methods developed for tomotherapy [20,21], we adopted a computer graphics approach to DRT generation, offering detector independence and speed.

Modeling imaging coordinate distortion is crucial for verification. While longitudinal scaling applies to both 2D and 3D imaging, 3DCT compression depended on the scan range and was modelled using high-precision laser measurements. Distortions in 3DCT were an order of magnitude higher than previously reported [5,22], likely due to scanner-specific factors or reference data accuracy. For topograms, a single scaling factor based on the distance from the parking position maintained clinical tolerances [23]. We attribute this to differences in the scanner mechanics and angular momentum from the spinning gantry during 3D tomography. Alternatively, embedded table markers could enable on-the-fly correction; however, table bending due to patient weight and limited reproducibility of insert docking hinders direct mapping to absolute coordinates, making this approach impractical.

Acquiring diagnostic-quality CT images of patients in their treatment position facilitates modern therapeutic approaches that combine positioning and treatment adaptation for optimal conformity [24]. A recent survey identified workflow complexity, QA demands, and high staffing requirement as key barriers to the clinical use of in-room CT in adaptive proton therapy (APT), while emphasizing the need for greater automation and integration [25]. Although previous studies on in-room CT-based APT have focused on plan comparisons [26,27], they often overlook a critical aspect of clinical translation: the accurate computation of treatment table positions from daily images to enable the delivery of adapted plans, which is specifically addressed in this work.

In conclusion, we presented the geometric calibration of an in-room CT-on-rails for high-precision radiotherapy, including a general approach to coordinate mapping for treatment table position calculation. With modality-specific distortion corrections, remote CT imaging achieves high accuracy and removes the need for verification imaging at the treatment isocenter.

## Declaration of competing interest

The authors declare the following financial interests/personal relationships which may be considered as potential competing interests: Antony John Lomax declares a research collaboration agreement with Siemens Healthineers related to CT imaging and its use in the context of image-guided radiation therapy and being member of Leo Cancer Care scientific advisory board. The other authors have no competing interests to disclose related to the current manuscript.
