# Supplementary material for: Topogram and 3DCT geometry calibration for image-guided proton therapy with in-room CT-on-rails
Source: Phys Imaging Radiat Oncol. 2025 Jun 24;35:100799. doi: 10.1016/j.phro.2025.100799 (PMC12269447; doi:10.1016/j.phro.2025.100799)
Supplement: Supplementary Data 1 [file mmc1.pdf]

Supplementary material

A. Treatment table bending calibration

The treatment table bends under load, with different deformations occurring between the CT imaging position and the treatment position due to variations in the configuration of the robot segments. To ensure that an isocenter identified on the CT images acquired with the table at the imaging position aligns with the room isocenter, it is necessary to calibrate such bending difference and apply corrections to the table position accordingly.

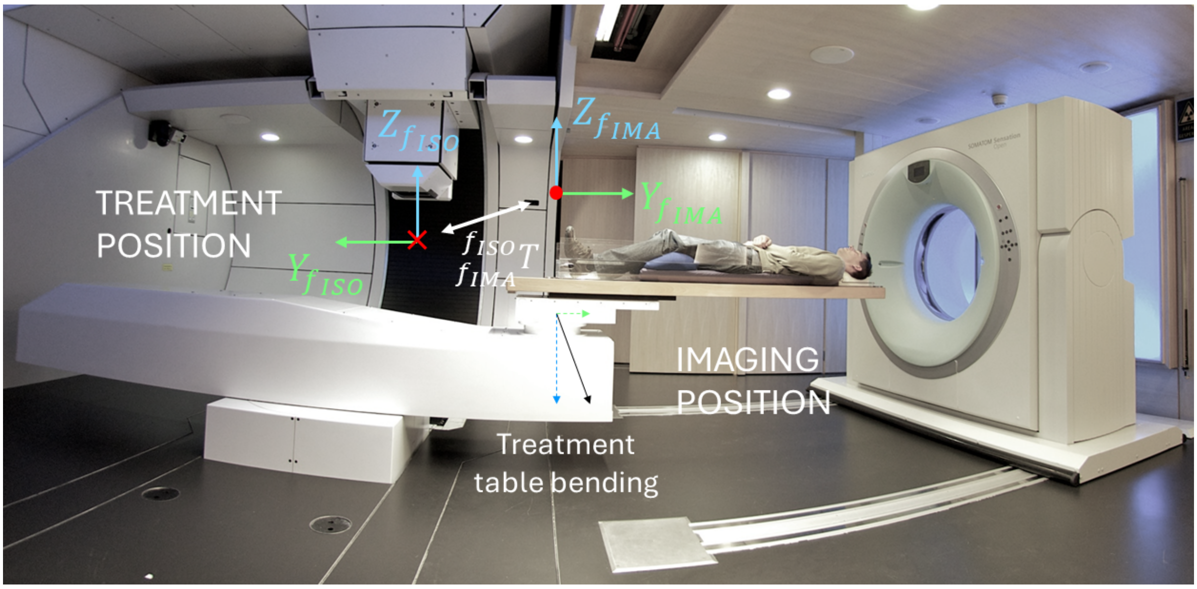

Figure S1: a photo of Gantry 2 at the Paul Scherrer Institute annotated with frames of reference relevant to the table bending calibration, namely the fixed room coordinates system at treatment isocenter fISO and its mapping to the imaging position fIMA. At imaging position, under load, the table bends moving forward and down.

To achieve this, a fixed reference system is defined at CT imaging position (f_{IMA}), arbitrarily centered 17.5 cm above the rotational axis of the robot's end effector (Figure S1). A fixed transform $\left({}^{f_{ISO}}_{f_{IMA}}T\right)$ maps its position relative to the room's frame of reference (f_{ISO}). The frame of reference of an ideal, non-bending treatment table ($\dot{s}$) coincides with f_{IMA} in the CT imaging position but deviates due to mechanical bending (s) of the table loaded with weight (Figure S2). Because the robot arms' configuration differs between the CT and treatment positions, the bending characteristics at the treatment position are also different. The goal of calibration is to characterize both deformations and derive a single correction that ensures the field isocenter defined in the CT position is accurately aligned with the room isocenter for treatment.

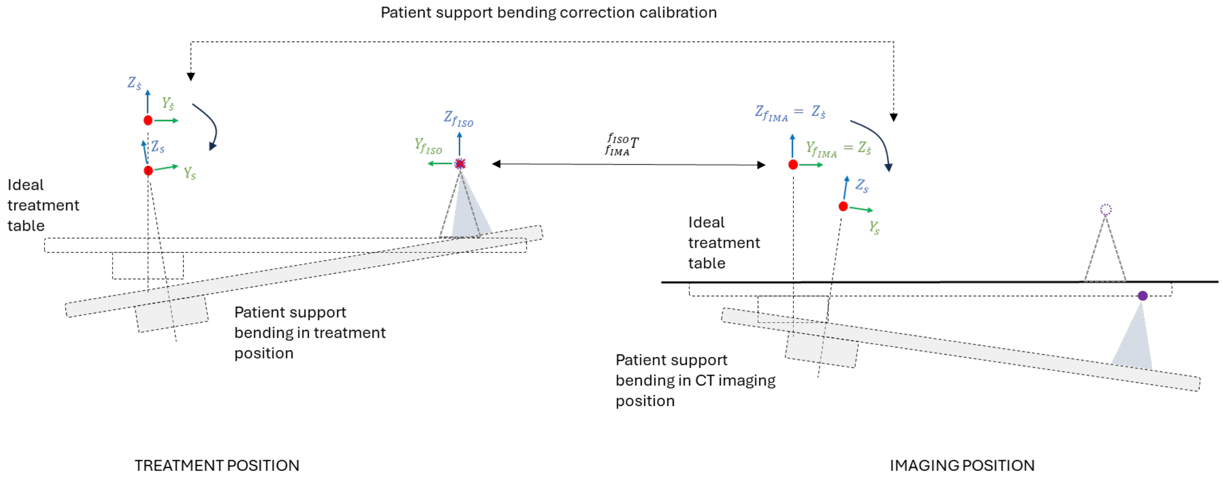

Figure S2: Schematic representation of the treatment table bending in imaging and treatment positions. Deformations are exaggerated for illustrative purposes.

This empirical calibration is achieved by comparing precise measurements of multiple reference points on an indexing plate on the table in both imaging and treatment positions (Figure S3). Reference points acquired relative to f_{IMA} are driven to the treatment isocenter f_{ISO} assuming an ideal patient support with no bending. The distance from the room isocenter f_{ISO} is evaluated to derive the correction of robot end effector that aligns the point.

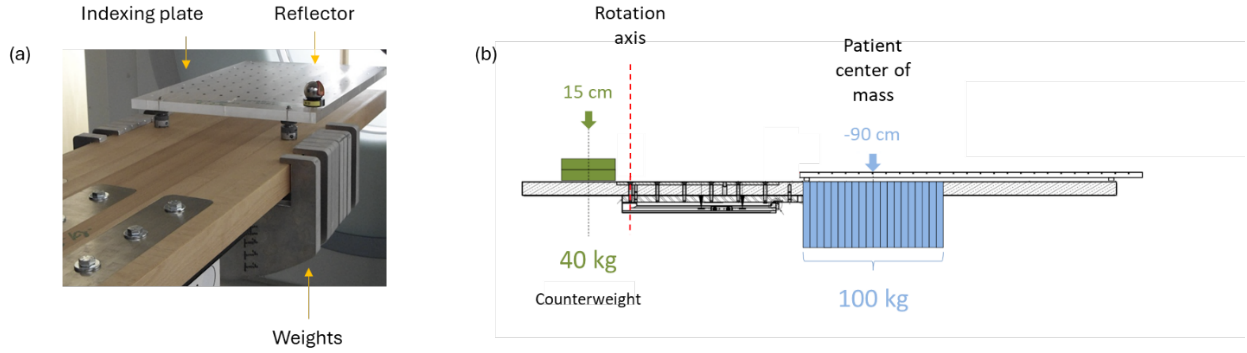

Figure S3: setup for table calibration measurements including an indexing plate to hold a laser tracker reflector (a) and metal plates that simulate the weight distribution of patients on the treatment table (b).

The process is repeated for every insert in clinical operation under different load conditions that simulate a range of patient weight classes, from infants to heavy adults, ensuring a comprehensive calibration that supports all scenarios. The correction is modeled using linear regression on measured data as a function of weight, torque, and position along the treatment table.

Calibration accuracy, assessed via verification measurements as the 3D distance between multiple table points positioned at isocenter and the room origin, was below 0.51 mm across all inserts, patient weight classes, and torque conditions (Figure S4).

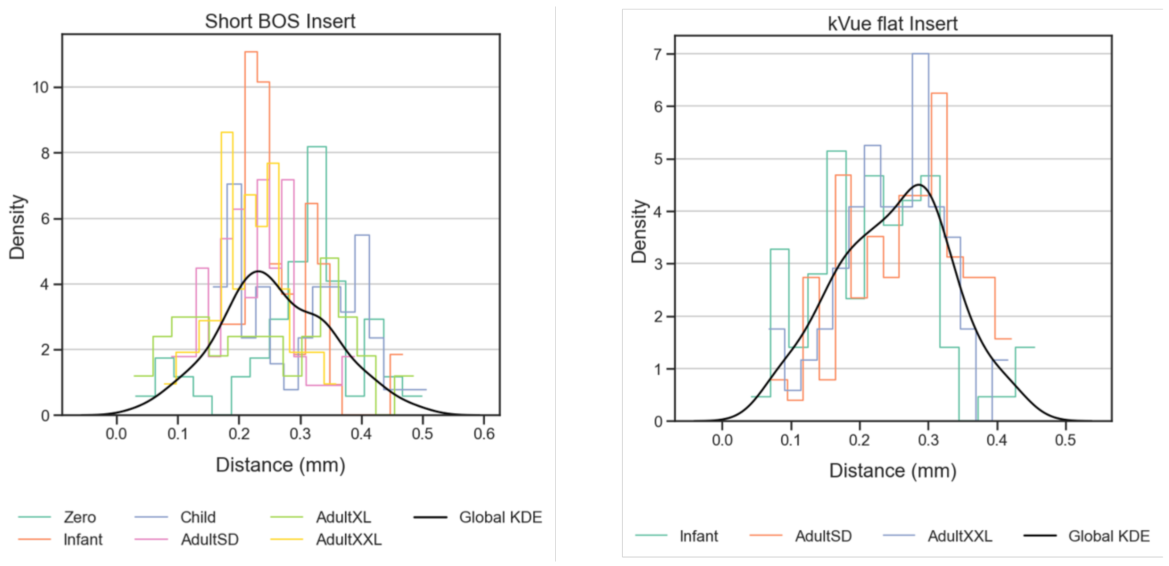

Figure S4: verification of treatment table bending calibration shown as 3D distance of multiple table points driven to the room coordinate origin and their nominal position, measured for two inserts and across different patient weight classes.

B. Phantoms

Geometric phantoms used to model the 3DCT imaging coordinates distortion and frame of reference calibration (Section 2.2) and to simulate clinical positioning (Section 3.4).

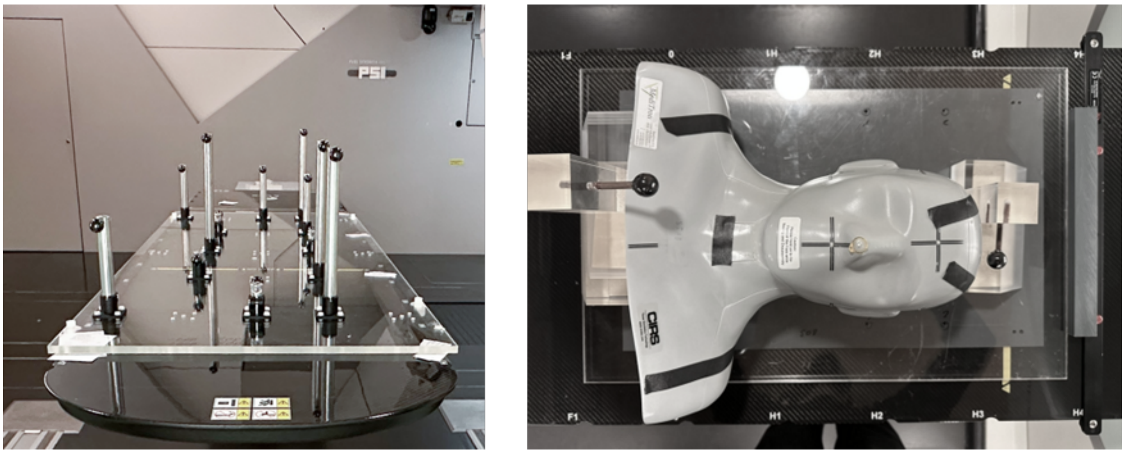

Figure S5: Left panel: 12-points phantom with three rows of spheres held at different levels from the table top; right panel: CIRS head phantom with two fiducial spheres at cranial and caudal positions.

C. Projective geometry for fan beam topogram imaging

The reconstruction of Digitally Reconstructed Topogram (DRT) images from 3DCT volumes is based on a modified version of Siddon-Jacobs algorithm for incremental ray tracing. The position of the source and each DRT pixel center can be defined to cast a ray for integration, extending twice the SID. Additionally, the conventional lateral dispersion of the cone beam has been replaced by a sliding source, with the X-ray source moving along its y-axis (CT_{S_y}, Figure S6) to align with the DRT pixels position.

Topogram projection: geometry definition

The fan beam projection begins with the transform mapping the CT frame of reference (FoR) and the X-ray source CT_S. For convenience, the CT origin is shifted to the edge of the image (CT_{IMA}), such as the pixels grow along positive axes (Figure S6).

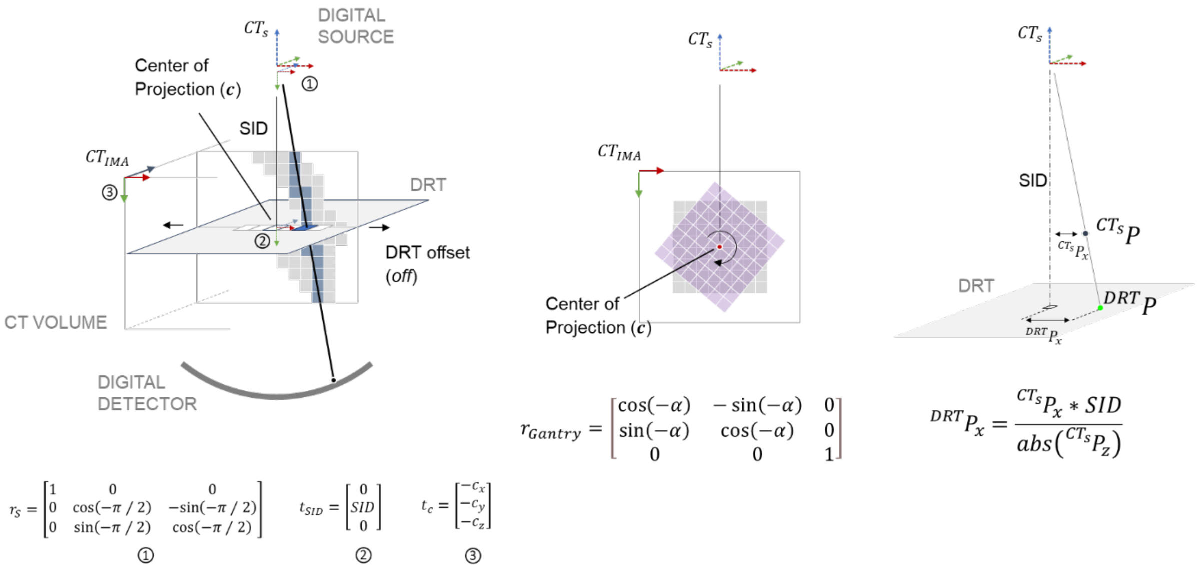

Figure S6: on the left, the projection geometry of digitally reconstructed images in standard conditions, with the source at 0° including (1) a rotation of the source frame of reference, (2) a translation by the SID and (3) by the image center. The gantry rotation (α) is simulated as inverse transform on the CT imaging grid (middle panel), which for illustratory purposes, is set to 45°. In-plane coordinates of projected points from the source can be obtained by correcting the lateral dispersion with intercept theorem from the SID, point's distance from the projection axis and source (right-panel).

For a projection geometry at $\alpha = 0°$ and head-first-supine patient orientation, such a transform is given by a -90° rotation of the source around x-axis, a shift by the SID distance and a final translation to the CT origin located $\boldsymbol{c}(c_x, c_y, c_z)$ away from the projection center. The resulting transform is therefore:

$$^{CT_{S(\alpha=0)}}_{CT_{IMA}}T = \begin{bmatrix} 1 & 0 & 0 & -c_x \\ 0 & \cos(-\pi/2) & -\sin(-\pi/2) & \mathrm{SID} - c_y \\ 0 & \sin(-\pi/2) & \cos(-\pi/2) & -c_z \\ 0 & 0 & 0 & 1 \end{bmatrix} \quad (Eq. 2)$$

The projection angle is introduced rotating the CT imaging grid by the inverse of the gantry (α). Such r_{Gantry} rotation has origin at the center of projection ($\boldsymbol{c}$):

$$^{CT_{IMA}}T_{Gantry} = \begin{bmatrix} r_{Gantry} & r_{Gantry} * \boldsymbol{c} - \boldsymbol{c} \\ 0 & 1 \end{bmatrix} \quad (Eq. 3)$$

Geometry calibration

In order to calibrate the projection geometry model, SID, $\boldsymbol{c}$ and α parameters are optimized minimizing the projection error on the DRT plane of 3DCT points against their position on actual topogram images. Any arbitrary position ($^{CT_{IMA}}P$) in the CT FoR can be mapped to the source ^{CT_S}P with $^{CT_S}_{CT_{IMA}}T = (^{CT_{IMA}}T_{Gantry} * \, ^{CT_{S(\alpha=0)}}_{CT_{IMA}}T)$, and afterwards, projected onto the DRT image plane by correcting for the fan beam lateral dispersion:

$$^{DRT}P = \begin{bmatrix} ^{CT_S}P_x * SID \Big/ abs(^{CT_S}P_z) \\ ^{CT_S}P_y \\ -SID \end{bmatrix} \quad (Eq. 4)$$

An offset (*off*) needs to be introduced in the DRT image after projection, i.e. $^{DRT}P'_x = \, ^{DRT}P_x + off$.

The calibration routine was implemented as constrained optimization using Broyden–Fletcher–Goldfarb–Shanno optimizer with a termination tolerance of 1e-13 using the CT-dots dataset (Section 2.3) and 24 fiducials (2 mm diameter) embedded in the table top. Each projection angle

and FoV was calibrated individually, ignoring the longitudinal direction (CT_{IMA_Z}, Figure S6), that is irrelevant for this calibration.

Patient alignment with 2D/3D topogram images registration

DRT generation has been integrated in a 2D/3D registration framework for Scout-guided RadioTherapy (ScoutRT) to compute the $T_{Patient}$ transform including translations ($t_{Patient}$) and rotations ($r_{Patient}$) that optimizes the similarity metric chosen between a pair of actual topogram images acquired at the scanner and the corresponding DRTs (Figure S7).

Depending on the registration settings, this is a six- or three-degree-of-freedom transform with origin at the treatment beam isocenter (${}^{CT_{IMA}}ISO$) as follows:

$$ {}^{CT_{IMA}}T_{Patient} = \begin{bmatrix} r_{Patient} & T_{Patient} * {}^{CT_{IMA}}ISO - {}^{CT_{IMA}}ISO \\ 0 & 1 \end{bmatrix} \quad (Eq. 5) $$

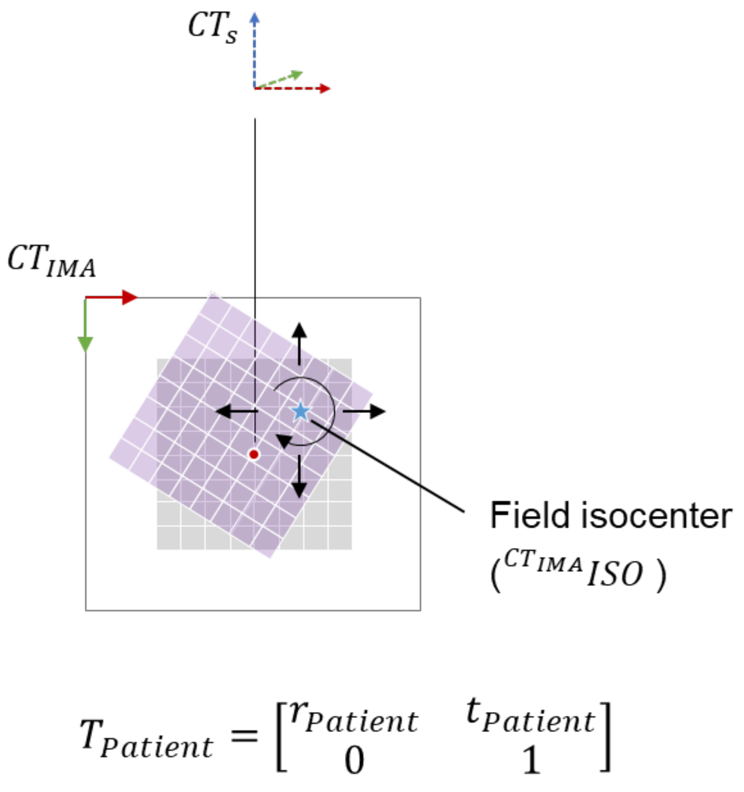

Figure S7: schematic representation of the patient transform for an arbitrary isocenter position defined in the CT_{IMA} frame of reference.

D. CT-on-rails imaging reproducibility

The reproducibility of CT imaging geometry was measured as variation of fiducials position in image coordinates. The 95% interval (97.5th – 2.5th percentile range) from the mean position is given for three 3DCT scan regions (R1-2-3) of the spheres auto-segmentations and manual localization of CT-dots in 3DCT and topogram imaging at two field of view (FoV).

		3D delta from mean [mm] (95% interquartile range)
**Spheres**		
3DCT		
	R1	0.08
	R2	0.15
	R3	0.11
**CT-dots**		
3DCT		0.39
*Topogram**		
	Large FoV	0.50
	Small FoV	0.28

* contribution from the two in-plane directions only

E. Calibration of 3DCT imaging coordinates

The distortion along the longitudinal axis coordinates was calculated by optimizing a point-based registration between the positions of the reference fiducials from laser tracker measurements and their corresponding position in the 3DCT imaging. The registration includes three datasets acquired at three different scan ranges, and in the optimization process an affine transform is applied to the y-coordinate, allowing for different scaling in each range. The fiducial registration error (FRE) with and without the application of longitudinal scaling is shown in Figure S8 for the complete set of 20 sphere markers (Figure S5).

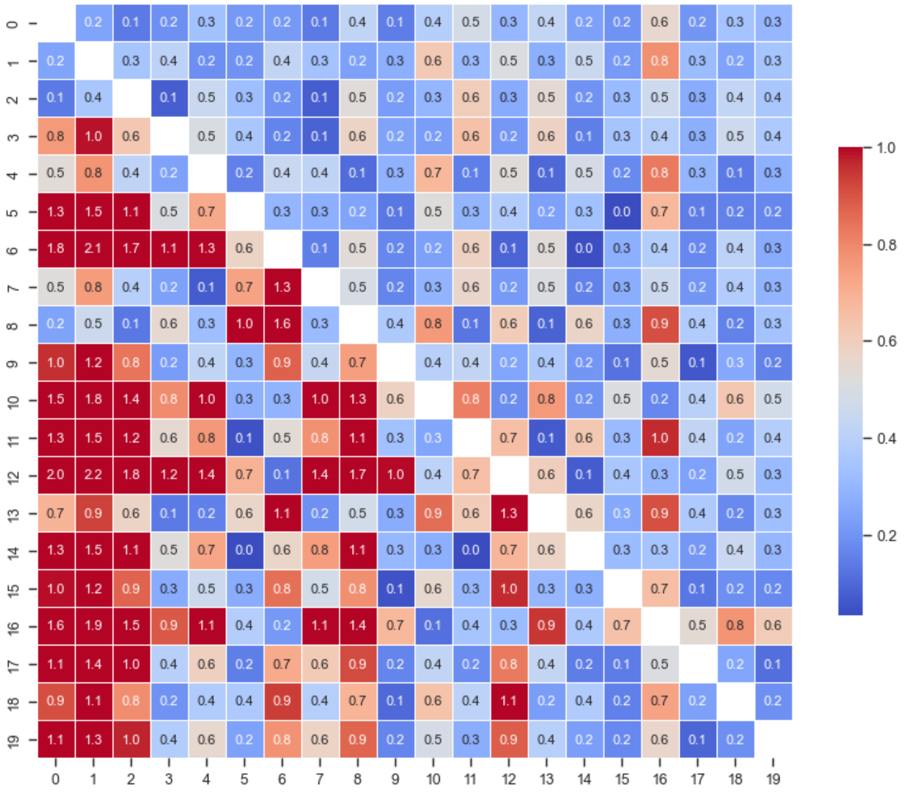

Figure S8: FRE heat map plot [mm] with (top-right) and without (bottom-left) scaling correction of imaging coordinates along the longitudinal direction of scanner motion. The red-blue transition threshold is set at 0.5 mm.

F. Topogram imaging projective geometry calibration parameters

Consistently with the projection geometry definition described in Section C, the topogram imaging geometry parameters for our scanner, a Siemens Sensation Open, are as follows – delta with respect to nominal:

	Nominal		HFS				FFS			
			Small FoV		Large FoV		Small FoV		Large FoV	
	LAT	PA	LAT	PA	LAT	PA	LAT	PA	LAT	PA
Angle [°]	90	180	0.49	0.21	0.72	0.28	0.49	0.75	0.77	0.76
SID [mm]	570	570	-0.33	-0.22	7.18	1.21	2.82	1.25	-0.84	0.86
Cx [mm]	0	0	0.59	0.06	1.42	-0.06	0.14	0.27	2.10	0.06
Cz [mm]	0	0	-0.35	-4.34	0.89	-2.92	0.09	1.45	-0.83	2.24
Off [mm]	0	0	-0.65	4.46	-0.40	2.12	-0.06	-1.37	-3.13	-3.19

G. Clinical simulation results

Residual errors following manual and automatic 2D/3D registration with CT topogram images or 3D-3D with in-room 3DCT images with respect to laser tracker reference measurements. Directions in IEC-world frame of reference.

Data dimensionality	Type and DoF	FoV		Δx [mm]	Δy [mm]	Δz [mm]
		PA	LAT			
2D/3D	Manual 3DoF	Small	Small	-0.18	-0.01	0.12
	Automatic 3DoF	Small	Small	-0.27	-0.01	-0.05
	Automatic 3DoF	Large	Large	-0.36	0.55	0.13
	Automatic 3DoF	Small	Large	-0.26	0.30	0.20
	Automatic 3DoF	Large	Small	-0.37	0.48	0.19
3D/3D	Automatic 6DoF	--		0.08	-0.10	0.15
	Manual 3DoF			-0.03	-0.09	0.09
